# Adherence to Antipsychotic Medication and Criminal Recidivism in a Canadian Provincial Offender Population

**DOI:** 10.1093/schbul/sbx084

**Published:** 2017-06-20

**Authors:** Stefanie N Rezansoff, Akm Moniruzzaman, Seena Fazel, Lawrence McCandless, Julian M Somers

**Affiliations:** 1 Somers Research Group, Faculty of Health Sciences, Simon Fraser University, Burnaby, BC, Canada;; 2 School of Social Welfare, University of California—Berkeley, Berkeley, CA;; 3 Department of Psychiatry, Warneford Hospital, University of Oxford, Oxford, UK;; 4 Faculty of Health Sciences, Simon Fraser University, Burnaby, BC, Canada

**Keywords:** medication possession ratio, public safety, criminal recidivism, antipsychotic adherence, adherence threshold

## Abstract

Preliminary evidence suggests that adherence to antipsychotic medication reduces criminal recidivism among patients diagnosed with schizophrenia. However, existing studies operationalize antipsychotic adherence as a binary variable (usually using a threshold of ≥80%), which does not reflect the prevalence of suboptimal adherence in real-world settings. The purpose of the current analysis was to investigate the association between successive ordinal levels of antipsychotic adherence and criminal recidivism in a well-defined sample of offenders diagnosed with schizophrenia (*n* = 11462). Adherence was measured using the medication possession ratio (MPR) and analyzed as a time-dependent covariate in multivariable regression models. Data were drawn from linked, comprehensive diagnostic, pharmacy and justice system records, and individuals were followed for an average of 10 years. Adjusted rate ratios (ARR) and confidence intervals (CI) are reported. Overall mean MPR was 0.41. Increasing levels of antipsychotic adherence were not associated with progressively lower rates of offending. However, when compared to the reference group (MPR ≥ 80%) all lower adherence levels were significantly associated (*P* < .001) with increased risk of violent (ARR = 1.58; 95% CI = 1.46–1.71) and nonviolent (ARR = 1.41; 95% CI = 1.33–1.50) offenses. Significance was replicated in separate sensitivity analyses. Previously published studies reporting reductions in crime may have been influenced by antipsychotic adherence ≥80%. Binary operationalization of adherence is an inaccurate predictor of recidivism. Future research addressing functional outcomes of antipsychotic adherence should conceptualize adherence as an incremental independent variable.

## Introduction

Antipsychotic medication is the cornerstone of treatment for schizophrenia and considered instrumental in the management of violent behavior. A groundbreaking study in a general population (published in 2014) reported a 45% reduction in violent crime among patients when they received antipsychotic medication compared to when they did not.^1^ A more recent analysis concluded that pharmacological treatment of schizophrenia significantly delayed time to violence by 18% among offenders released from prison.^2^ Nonetheless, the relationship linking violence and psychosis is unclear.^3^

A limitation of existing research involves the definition of adherence. Although routinely operationalized in binary terms (ie, adherent vs nonadherent), adherence to antipsychotics varies considerably,^4^ and the term “partially adherent”^5^ may best describe the status of most patients when observed over time. Previous research addressing violent offending^1^ applied an innovative within-participant analytic approach, but nevertheless investigated adherence in binary terms, concluding that any adherence (at least one prescription within 120 days) was superior to none.

Clinical practice guidelines often recommend adherence levels at or above 80%^6^: the cut-off point attributed to a 1975 definition of adherence involving antihypertensive medication.^7^ Empirical evidence in support of this threshold is limited,^8^ although it is widely used in drug adherence research,^9^ including studies focused on antipsychotics. Very few studies report multiple levels of adherence from 0% to 100%,^10^ and the definition of “poor adherence” (vs nonadherence) is obscure.^11^

Preliminary evidence suggests that ≥80% antipsychotic adherence is associated with lower criminal justice involvement and related costs.^12,13^ This research represents important groundwork addressing the relationship between adherence and criminality, but is subject to methodological constraints, including the use of self-reported criminal history, relatively short periods of follow-up, and heterogeneous samples (ie, hospitalized psychiatric patients and individuals eligible for Medicaid). These limitations are problematic, and the validity of self-reported justice-system involvement is particularly low.^14^ Additional research addressing the relationship between adherence and crime (ie, violent *and* nonviolent offending) has been explicitly recommended.^15^

The current study addresses this recommendation, using comprehensive administrative records from a well-defined offender population diagnosed with schizophrenia and adjudicated in British Columbia (BC, Canada). We compared guideline level adherence (≥80%) with lower levels of adherence (ie, ≤19%; 20%–39%; 40%–59%; 60–79%). Participants were followed over a mean observation period of nearly 10 years.

Our primary hypothesis was that antipsychotic adherence at or above the 80% threshold would be associated with a significantly reduced rate of violent and nonviolent convicted offenses, when compared to lower levels of adherence. To examine whether higher levels of adherence were associated with incrementally less offending, we tested the secondary hypothesis that the lowest level of adherence (0%–19%) would be associated with the highest rate of criminal recidivism.

## Methods

### Data Sources

The study was approved by the Research Ethics Board at Simon Fraser University. Analyses were conducted using data from the BC Inter-Ministry Research Initiative (IMRI), which integrates linked, nonidentifying administrative data spanning multiple decades from 3 independent BC government ministries: 1990–2015 from the Ministry of Health (MoH), and 1997–2015 from the Ministries of Justice (MoJ) and Social Development. The database includes individual-level records related to diagnoses, hospital and outpatient services, prescription medications, income assistance, justice encounters, and sociodemographic variables. Details of the IMRI database not essential to the current study have been described elsewhere.^16–18^

The current analysis used 3 specific data sets: Medical Services Plan (MSP) billing data and BC PharmaNet from the MoH, and convicted offense data from the MoJ. The MoJ administers sentences for all provincial offenders. MSP data represent medical services delivered to all residents of BC, including dates, costs and diagnostic codes (based on International Classification of Diseases: ICD-9). Participation in MSP is mandatory and comprises the single source of payment for publicly administered medical services in BC.

All prescriptions dispensed in BC are recorded in BC PharmaNet, which has been shown to accurately reflect medication adherence for most patients.^19^ These data are sufficiently detailed to calculate long-term adherence to prescription medications and have previously been used to assess antipsychotic adherence.^20,21^ IMRI data, including PharmaNet records, cover periods of time when offenders were incarcerated, as well as all available time before and after involvement with the criminal justice system. Financial disincentive against the use of prescribed medications is minimized in BC as the out-of-pocket cost is incrementally linked to income. Individuals with low incomes are eligible for publicly funded prescription coverage. Antipsychotic medications were identified using the American Hospital Formulary Service List.

### Study Design and Participants

The population available for analysis consisted of all individuals convicted under BC jurisdiction between January 1, 1998 and March 31, 2015. We only included those offenders diagnosed with schizophrenia (ICD-9; code 295), and following the convention used in previous research,^1^ for whom we had at least 120 days of follow-up. A diagnosis of schizophrenia at any time during the study period was used to identify individuals eligible for the study. All diagnoses were made by licensed practitioners in British Columbia. Clinicians were identified as psychiatrists, general practitioners, and nurses/allied health professionals to allow for diagnostic comparison based on practitioner specialty.

Because comprehensive Justice records are only available for individuals 18 years of age and older, we excluded those prescribed antipsychotics prior to the age of 18. Offenders were followed from the date of first dispensed antipsychotic prescription until censoring (date of death or March 31, 2015).

### Variables of Interest

Adherence to antipsychotic prescription was our primary independent variable, operationalized using the medication possession ratio (MPR). MPR represents the percentage of time that an individual was dispensed prescribed medication (ie, the number of days of medication supplied within a refill interval divided by the total number of days in the interval), and is the preferred measure of adherence using administrative data.^22^ Antipsychotic MPR has previously been correlated with likelihood of arrest among adults with serious mental illness.^12,13^

Following Fazel et al,^1^ we calculated adherence (in our case, MPR) for each 120-day interval, beginning with the date of antipsychotic medication initiation. Antipsychotic polypharmacy on any given day did not result in double counting or inflation of the MPR (ie, upper bound = 1.0 per day). Although participants may have received more than one antipsychotic agent or varying doses over time, the current analysis did not focus on changes in medication regimen during the follow-up period. MPR was categorized into 5 groups (≤0.19; 0.20–0.39; 0.40–0.69; 0.60–0.79; ≥0.80) and analyzed as a time-dependent covariate in the regression model. Consistent with existing literature, MPR ≥0.80 was used as the reference group.

Our primary outcome of interest was the number of convicted offenses recorded in each 120-day interval for all participants in the cohort. These were classified as violent or nonviolent based on the type of offense (specified). The date of the offense was the date of the outcome. In cases where the date of offense was not reported, this was approximated using the date of conviction and the average latency of 5 months between the date of offense and date of conviction.

### Statistical Analyses

We used generalized estimating equations (GEE) to estimate the effect of MPR on the rate of violent and nonviolent crime,^23^ while adjusting for confounding variables. We selected GEE negative binomial models (negative binomial distribution with log link) due to the over-dispersion and count nature of the outcome data, and for the better goodness of fit statistics relative to Poisson regression. Autoregressive (first-order) correlation structure was chosen to control for the correlation in repeated measures of the number of offenses. As an additional safeguard against heteroskedasticity and potential misspecification, we used the robust variance estimator to estimate standard errors for the parameters.^24–27^ In the GEE negative binomial regression analysis, we initially estimated the dispersion parameter ignoring the correlation between individuals, and then imputed the value of the dispersion parameter in the model, as suggested by Hilbe.^26^

Each multivariable model controlled for several variables selected a priori as potential confounders associated with criminal behavior. These include: patient age (centered; age 18), gender (men and women), ethnicity (White, Indigenous and other), education level (<Gd. 10, Gd. 10/11, Gd. 12, and Vocational/University), frequency of substance use-related services in each 120-day interval (continuous variable), frequency of prior convicted offenses in each 120-day interval (continuous variable), and duration of follow-up in days (offset variable). We report unadjusted and adjusted rate ratios along with 95% confidence intervals as measures of association (effect sizes). Significance of estimated parameters is reported using alpha level (*P* ≤ .05), and all *P* values are 2-sided.

Cases with missing values were included in the primary analysis. Demographic information was missing for 1280 (11%) of participants: age, *n* = 3(<1%); ethnicity, *n* = 573 (5%); and education level, *n* = 1193 (10%). These participants were included using mean age as well as “unknown ethnicity” and “unknown education level” as separate categories in the multivariable model.

### Sensitivity Analyses

Sensitivity analysis was performed to compare results of the primary analysis to those based on complete cases (*n* = 10182). Further sensitivity analyses were conducted: (*a*) using standard follow-up times of: <5 years, 5–<10 years, and ≥10 years; (*b*) restricted to surviving participants only (*n* = 10514); and (*c*) based on the origin of diagnosis (psychiatrist vs nonpsychiatrist). IBM SPSS Statistics 23^28^ and STATA 13^29^ were used to conduct all analyses.

## Results

### Sample Characteristics

**Fig. 1.**
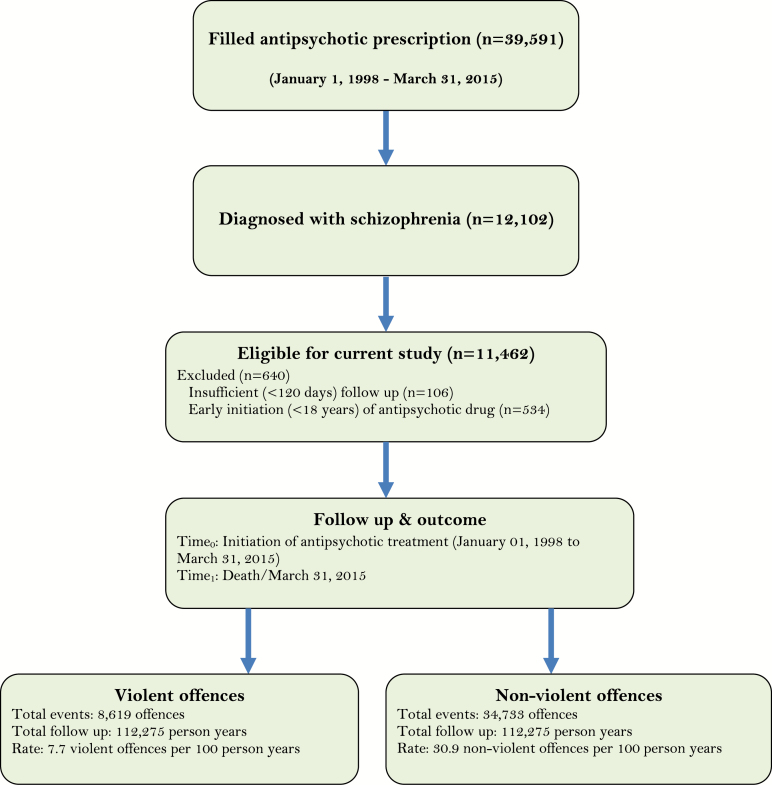
Flow chart of offenders included in the study.

Figure 1 illustrates the flow of patients included in the current study. Between January 1998 and March 2015, 39591 offenders convicted under BC provincial jurisdiction received at least one antipsychotic prescription. Of these individuals, 31% (*n* = 12102) were diagnosed with schizophrenia (the majority—71%—were diagnosed by psychiatrists and the remainder by other specialists). We excluded 640 offenders due to insufficient follow-up time (<120 days) and/or initiation of an antipsychotic prescription prior to age 18. In total, 11462 individuals met our inclusion criteria.

### Randomized

Offenders in the study sample were predominantly men, with a mean age of 35 years at enrollment (table 1). The majority identified ethnically as White, and 11% were of self-reported Indigenous ethnicity. Roughly half of the sample (48%) had completed grade 12 and/or vocational or postsecondary training.

**Table 1.** Sociodemographic Characteristics of the Study Population (*n* = 11462)

Variable	Mean (SD)/ *n* (%)
Age at enrollment^a^	
Mean (SD)	35.2 (11.0)
Median (IQR)	34.1 (26.2, 42.6)
Men, *n* (%)	9022 (78)
Ethnicity, *n* (%)	
White	8210 (72)
Indigenous	1289 (11)
Other	1390 (12)
Unknown	573 (5)
Education level, *n* (%)	
<Grade 10	1613 (14)
Grade 10/11	3205 (28)
Grade 12	3694 (32)
Vocational/University	1757 (16)
Unknown	1193 (10)

*Note*: IQR, inter-quartile range; SD, standard deviation.

^a^Age at enrollment was based on the date of initiation of antipsychotic treatment (between January 01, 1998 and March 31, 2015).

### Antipsychotic MPRs

Antipsychotic adherence and crime-related characteristics of the study sample are provided in table 2. The mean follow-up period approached 10 years, and overall MPR was 0.41. The percentage of participants who met or exceeded the recommended 0.80 adherence threshold dropped from 35% in the first 120 days of follow-up to 27% in the subsequent 120-day interval. Concurrently, the percentage with MPR below ≤0.19 increased from 21% to 46%, indicating that initial adherence levels were not sustained.

**Table 2.** MPR and Crime-Related Characteristics of the Study Population (*n* = 11462)

Individual-Level Variable	Mean (SD) / *n* (%)
Number of days with antipsychotic medication in follow-up period	
Mean (SD)	1475 (1544)
Median (IQR)	883 (210, 2334)
Follow-up period, in years	
Mean (SD)	9.8 (5.1)
Median (IQR)	9.7 (5.4, 14.3)
Minimum, maximum	0.3, 17.2
Total follow-up time (person years)	112, 275
MPR (during entire follow-up period)	
Mean (SD)	0.41 (0.32)
Median (IQR)	0.36 (0.09, 0.70)
MPR (during 1st 120-day interval of follow-up period), n (%)	
≤0.19	2392 (21)
0.20–0.39	2052 (18)
0.40–0.59	1435 (12)
0.60–0.79	1628 (14)
≥0.80	3955 (35)
MPR (during 2nd 120-day interval of follow-up period), *n* (%)	
≤0.19	5222 (46)
0.20–0.39	1001 (9)
0.40–0.59	955 (8)
0.60–0.79	1197 (10)
≥0.80	3087 (27)
No. of offenses in the year prior to enrollment, mean (SD)	0.8 (1.9)
Any offense in the year prior to enrollment, *n* (%)	3341 (29)
Violent offenses in follow-up period	
Mean no. of offenses (SD)	0.8 (1.8)
Median no. of offenses (IQR)	0 (0, 1)
Total no. of violent events in the entire cohort	8619
Rate (per 100 person years)	7.7
Nonviolent offenses in follow-up period	
Mean no. of offenses (SD)	3.0 (7.3)
Median no. of offenses (IQR)	1 (0, 2)
Total no. of nonviolent events in the entire cohort	34733
Rate (per 100 person years)	30.9

*Note*: IQR, inter-quartile range; SD, standard deviation; MPR, medication possession ratio.

The vast majority (87%) of prescribed antipsychotics were atypical (supplementary table 1). Of these, quetiapine (39%), olanzapine (19%), and risperidone (17%) were the most common. Only 7% of prescriptions were for clozapine. Loxapine and methotrimeprazine were the most commonly prescribed typical antipsychotics.

### Justice Outcomes

Convicted violent offenses totaled 8619 throughout follow-up; averaging nearly one (0.8) violent offense per person during the study period, or a rate of 7.7 violent offenses per 100 person years. The rate of nonviolent offending was 4 times higher (30.9 per 100 person years), averaging 3 nonviolent offenses per person, and totaling 34733 during follow-up. Although the overall mean number of convicted offenses in the year prior to enrollment approached one (0.80) per person, these were committed by less than one-third (29%) of the cohort. Categories and frequencies of violent and nonviolent crime are provided in supplementary table 2.

### GEE Negative Binomial Regression Analysis

Crude and adjusted rate ratios (ARR) and 95% confidence intervals (CI) are reported for each level of adherence compared to the reference (ie, ≥0.80; table 3). Each of the lower adherence quintiles was associated with significantly (*P* < 0.001) higher rates of both violent and nonviolent offending. The lowest level of MPR (≤0.19) was associated with the lowest mean rate of violent (ARR: 1.39; CI: 1.27–1.52) and nonviolent (ARR: 1.31; CI: 1.21–1.41) offending among the 4 comparison levels. The effect of antipsychotic adherence was consistently greater for violent offenses. Restricting the sample to complete cases (ie, those with complete information across all databases; *n* = 10182) and repeating the above analysis revealed a similar pattern of findings (supplementary table 3).

**Table 3.** GEE Negative Binomial Regression Analysis Estimating the Association Between Medication Possession Ratio (MPR) and Violent/Nonviolent Crime (*n* = 11462^a^)

	Variable	Unadjusted Rate Ratio	Adjusted Rate Ratio
		(95% CI)^b^	(95% CI)^c^
Violent offenses	MPR		
	≤0.19	**1.54 (1.41, 1.68**)^**d**^	**1.39 (1.27, 1.52**)
	0.20–0.39	**2.68 (2.39, 3.01**)	**2.28 (2.03, 2.56**)
	0.40–0.59	**2.58 (2.29, 2.91**)	**2.21 (1.96, 2.50**)
	0.60–0.79	**1.95 (1.74, 2.19**)	**1.73 (1.55, 1.94**)
	≥0.80	Reference	Reference
	MPR (<0.80)	**1.78 (1.64, 1.93**)	**1.58 (1.46, 1.71**)
Nonviolent offenses	MPR		
	≤0.19	**1.50 (1.39, 1.62**)	**1.31 (1.21, 1.41**)
	0.20–0.39	**2.01 (1.83, 2.21**)	**1.64 (1.50, 1.80**)
	0.40–0.59	**1.86 (1.70, 2.03**)	**1.64 (1.50, 1.80**)
	0.60–0.79	**1.65 (1.53, 1.78**)	**1.48 (1.37, 1.60**)
	≥0.80	Reference	Reference
	MPR (<0.80)	**1.62 (1.52, 1.73**)	**1.41 (1.33, 1.50**)

*Note*: GEE: generalized estimating equation; MPR: medication possession ratio; CI: confidence interval.

^a^Demographic information was unknown for 1280 participants (11%): age *n* = 3 (<1%); ethnicity *n* = 573 (5%); and education level *n* = 1193 (10%). Unknown age was replaced by mean age. Unknown ethnicity and education level were included as separate categories in the multivariable model.

^b^95% CIs and both unadjusted and adjusted rate ratios were estimated using robust standard errors.

^c^Each multivariable GEE model controlled for age at enrollment (centered, age 18), gender (men and women), ethnicity (White, Indigenous, other, and unknown), education level (<Gd. 10, Gd. 10/11, Gd. 12, Vocational/University, and unknown), use of substance disorder-related services (continuous variable), number of offenses in the previous year (continuous variable), number of 120-day intervals (continuous variable), and duration of follow-up in days (offset variable).

^d^Bold indicates significant at *P* value <.001.

The relationship between MPR and violent/nonviolent offending was also subject to sensitivity analysis by duration of available follow-up. Table 4 demonstrates that MPR levels ≤0.79 were each associated with significantly higher rates of both violent (*P* < .001) and nonviolent (*P* < .05) offending compared with the reference group, regardless of the length of follow-up. Similar overall results were generated when individuals who died during follow-up were excluded from analyses. Sensitivity analysis estimating the association between MPR and violent/nonviolent offenses by diagnostic source (psychiatrist vs nonpsychiatrist) also yielded the same pattern of findings (supplementary table 4).

**Table 4.** Sensitivity Analysis Estimating the Association Between Medication Possession Ratio (MPR) and Violent/Nonviolent Crime (by Death and Duration of Follow-Up)

Subgroup	MPR	Violent Crime	Nonviolent Crime
		ARR^a^ (95% CI^b^)	ARR (95% CI)
Surviving participants (*n* = 10514,^c^ 92%)			
	≤0.19	**1.35 (1.23, 1.48**)^d^	**1.22 (1.22, 1.42**)
	0.20–0.39	**2.29 (2.03, 2.58**)	**1.63 (1.49, 1.79**)
	0.40–0.59	**2.26 (1.99, 2.56**)	**1.65 (1.50, 1.81**)
	0.60–0.79	**1.70 (1.51, 1.91**)	**1.47 (1.36, 1.60**)
	≥0.80	Reference	Reference
	MPR (<0.80)	**1.55 (1.43, 1.68**)	**1.41 (1.33, 1.51**)
<5 years of follow-up (*n* = 2530, 22%)			
	≤0.19	**1.57 (1.18, 2.09**)	**1.44 (1.17, 1.78**)
	0.20–0.39	**2.31 (1.55, 3.44**)	**1.69 (1.29, 2.22**)
	0.40–0.59	**2.18 (1.46, 3.27**)	**1.40 (1.08, 1.80**)
	0.60–0.79	**1.89 (1.20, 2.96**)	***1.29 (1.00, 1.65***)^e^
	≥0.80	Reference	Reference
	MPR (<0.80)	**1.77 (1.35, 2.32**)	**1.43 (1.19, 1.72**)
5–<10 years of follow-up (*n* = 3395, 30%)			
	≤0.19	**1.48 (1.24, 1.76**)	**1.28 (1.13, 1.45**)
	0.20–0.39	**2.27 (1.84, 2.81**)	**1.51 (1.29, 1.78**)
	0.40–0.59	**2.16 (1.71, 2.72**)	**1.59 (1.36, 1.86**)
	0.60–0.79	**1.67 (1.34, 2.08**)	**1.41 (1.23, 1.61**)
	≥0.80	Reference	Reference
	MPR (<0.80)	**1.64 (1.40, 1.92**)	**1.37 (1.23, 1.53**)
≥10 years of follow-up (*n* = 5537, 48%)			
	≤0.19	**1.36 (1.22, 1.51**)	**1.31 (1.18, 1.44**)
	0.20–0.39	**2.28 (1.97, 2.65**)	**1.70 (1.50, 1.91**)
	0.40–0.59	**2.24 (1.93, 2.61**)	**1.69 (1.50, 1.91**)
	0.60–0.79	**1.74 (1.52, 2.00**)	**1.53 (1.38, 1.69**)
	≥0.80	Reference	Reference
	MPR (<0.80)	**1.55 (1.41, 1.71**)	**1.43 (1.32, 1.55**)

*Note:* ARR, adjusted rate ratio; CI: confidence interval; MPR, medication possession ratio.

^a^Each multivariable GEE model controlled for age at enrollment (centered, age 18), gender (men and women), ethnicity (White, Indigenous, and other); education level (<Gd. 10, Gd. 10/11, Gd. 12, and Vocational/University), use of substance disorder-related services (continuous variable), number of offenses in the previous year (continuous variable), number of 120-day intervals (continuous variable), and duration of follow-up in days (offset variable).

^b^95% CIs for the adjusted rate ratios were estimated using robust standard errors.

^c^948 participants (8%) who died during the study period were excluded from the analysis.

^d^Bold indicates significant at *P* value <.001.

^e^Italics bold indicates significant at *P* value ≤.05.

## Discussion

Antipsychotic adherence at or above an MPR of 0.80 was associated with a significantly lower risk of nonviolent offending (up to 65%), and violent offending (up to 128%) when compared to all lower levels of adherence. To our knowledge, this is the first longitudinal population-level study to investigate the association between multiple levels of antipsychotic adherence and convicted offenses.

Contrary to our secondary hypothesis, the lowest adherence level (MPR ≤ 0.19) was *not* associated with a relatively high offense rate. Although symptom improvement and/or remission (leading to treatment discontinuation) could explain the association between low MPR and comparatively low rates of offending, existing studies suggest that antipsychotic persistence is enhanced by effective symptom control.^30,31^

Increasing levels of adherence (ie, from 0.00 to 0.79) were not associated with progressive decreases in rates of offending. Clinical practices that achieve relatively high levels of adherence may have a more marked effect on crime reduction than practices intended to achieve incremental improvements in adherence below the 0.80 level. Of note, we observed relatively less criminal activity among patients in the lowest tier of adherence (ie, ≤0.19), which may have been the result of severe symptomatology (and associated impaired function) and/or hospitalization. Alternatively, titration from medication due to symptom improvement and recovery may account for the observed relationship between low adherence and offending in these patients. Further research is needed to establish the relationship between levels of adherence, patient functioning, and crime. In addition, research is needed to investigate the relationship between levels of adherence and a broad range of additional outcomes (eg, psychiatric symptoms, side effects, etc.).

Our results are consistent with those of the PRIDE study,^32^ in which the use of long-acting injectable (LAI) antipsychotics was associated with longer time to treatment failure (including arrest and incarceration) when compared to oral formulations. Authors of the study attributed these results to more consistent treatment exposure (ie, higher rates of adherence) in participants treated with injectables; a conclusion reiterated by authors of a second randomized controlled trial published in the same year,^33^ and further reinforced by research demonstrating superior psychopathological outcomes associated with “full” adherence (100%) when compared to “non-full” adherence (80%–99.9%) to both oral and depot antipsychotics.^34^ Over an average follow-up period of 10 years, mean MPR in our offender cohort was about half (0.41) the recommended level. Efforts targeted at reaching ≥80% adherence thus present an achievable opportunity for substantial crime reduction and meaningful violence prevention. Moreover, criminal justice involvement is strongly associated with negative health outcomes for offenders,^35^ and has distinct consequences for public health.^36^ Given the relatively high prevalence of schizophrenia among justice-involved individuals, the promotion of high antipsychotic adherence may also reduce a range of harms experienced by an already vulnerable population.

A further observation of note is that adherence achieved in the first 120 days of our study was not sustained in the subsequent 120-day period. Viewed chronologically, the first observed time interval had the highest proportion of offenders (35%) at or above 80% adherence. In the second interval, this proportion had declined to 27%. This is consistent with existing—but limited—research demonstrating that adherence to antipsychotic medication is unstable over time.^37^ Although current practice guidelines recommend “indefinite and continuous” antipsychotic treatment,^38^ discontinuation is common and is associated with extremely high risk of relapse.^39^

The significant reduction in offending associated with ≥0.80 adherence to medication demonstrated in the current study reinforces the importance of identifying and building on effective practices used during the first months of prescription to achieve sustained adherence over the longer term—particularly among patients involved with the criminal justice system.

### Strengths and Limitations

The current study extends existing research on the validity of 80% as a meaningful adherence threshold for antipsychotic medications and addresses a number of methodological constraints in previous studies (including the use of self-reported criminal history,^12^ binary adherence measurement,^1^ and relatively short periods of follow-up.^40^ We examined comprehensive administrative records from a large and well-defined population of offenders diagnosed with schizophrenia, spanning a mean observation period of nearly 10 years. Our study contributes novel outcome analysis by comparing guideline level adherence (≥80%) with lower levels (ie, ≤19%; 20%–39%; 40%–59%; 60–79%).

Nonetheless, our research has some limitations. Although MPR based on pharmacy records is a useful measure of adherence, it may overestimate actual ingestion of prescribed medication. Moreover, our dataset does not include the specific dosage prescribed to participants, and further research is needed investigating whether dose plays a role in promoting adherence and in reducing the risk of criminal recidivism. For reference, the strengths of all (oral) antipsychotic medications dispensed in British Columbia are presented in supplementary tables 5 and 6.

Medications administered in the hospital are not recorded in PharmaNet, and our analysis did not consider time at risk of offending (ie, time incarcerated). However, recent assessment of the BC offender population (*n* = 188625) demonstrated mean hospitalization of just 4.2 days, and mean custody convictions of merely 0.5 over a 5-year period (April 2007–March 2012).^16^

Although we maximized the use of available data by operationalizing MPR as a ratio, collapsing all prescribed antipsychotics into a single group potentially masked important variability between different medications and/or methods of administration (eg, LAIs), each of which warrants further research. Categorization of MPR in quintiles revealed important information about varying levels of adherence and our results lend support to the use of the 80% adherence threshold in scientific and clinical literature. Future research is needed to investigate the long-term associations between different levels of adherence and particular outcomes, specific formulations, and individual patient characteristics.

Existing literature suggests that untreated schizophrenia is associated with greater risk of police contact^41^ and violence.^42^ Large, longitudinal cohort studies have demonstrated significant reductions in violent recidivism among individuals when they were dispensed antipsychotic medication compared to when they were not.^1,43^ Nevertheless, a causal relationship between antipsychotic adherence and criminality cannot be inferred from the available evidence, and considerable debate remains over the relative importance of criminogenic factors vs psychotic symptoms in the treatment of mentally disordered offenders. Specifically, important correlates of criminal behavior, including personality traits (eg, impulsivity/aggression, poor self-control) and undiagnosed concurrent mental illness (eg, fetal alcohol spectrum disorders, personality disorders) were unaccounted for in the current multivariable model and may have resulted in residual confounding. Our findings may also have been subject to compliance bias, as treatment adherence captures unmeasured behaviors that may also lead to better patient outcomes (eg, willingness to comply with prescribed treatment, consistent engagement with health services and better patient insight).

## Conclusions

The current study is the first to examine the full range of antipsychotic medication adherence in an offender population spanning multiple years and found that adherence above 80% was associated with a significantly reduced risk of offending compared to all lower levels of adherence. Investigation of the links between adherence, violence, and offending among patients diagnosed with schizophrenia is indicated using experimental designs and investigating measures to achieve high levels of adherence.

Our findings provide empirical evidence of the effectiveness of antipsychotic medication for reducing both violent and nonviolent offending when adherence levels meet or exceed the 0.80 threshold. Further investigation addressing the association between different levels of antipsychotic adherence and diverse relevant clinical and social outcomes is required to inform optimal prescribing practices and objectives for patients with schizophrenia, and to set the stage for more personalized approaches to pharmacotherapy.

## Supplementary Material

Supplementary data are available at *Schizophrenia Bulletin* online.

## Funding

S.N.R. receives funding from the Canadian Institutes of Health Research (MFE148262). S.F. receives research funding from Wellcome Trust (095806). J.M.S. receives research funding from the Canadian Institutes of Health Research (SCT - 151611), the British Columbia Ministry of Justice (2014s0040), and Health Canada (2009s0231).

## Supplementary Material

Supplementary_Tables
