# Supplementary material for: Adherence to Antipsychotic Medication and Criminal Recidivism in a Canadian Provincial Offender Population
Source: Schizophr Bull. 2017 Jun 20;43(5):1002–10. doi: 10.1093/schbul/sbx084 (PMC5581906; doi:10.1093/schbul/sbx084)
Supplement: Supplementary_Tables [file sbx084_suppl_Supplementary_Tables.doc]

**Supplementary Table 1: Generic names of antipsychotic drugs (APD) prescribed to participants (n=11,462) during the study period**

|  | **Generic name** | **# of pharmacy transactions n (%)[[1]](#footnote-2)** |
| --- | --- | --- |
| **Atypical APD** | ARIPIPRAZOLE | 63,700 (2.1) |
|  | ASENAPINE MALEATE | 1,612 (0.1) |
|  | CLOZAPINE | 203,508 (6.8) |
|  | OLANZAPINE | 563,920 (19.0) |
|  | PALIPERIDONE | 11,280 (0.4) |
|  | PALIPERIDONE PALMITATE | 18,378 (0.6) |
|  | QUETIAPINE FUMARATE | 114,7367 (38.6) |
|  | RISPERIDONE | 51,8197 (17.4) |
|  | RISPERIDONE MICROSPHERES | 43,352 (1.5) |
|  | ZIPRASIDONE HCL | 24,909 (0.8) |
|  | LURASIDONE HCL | 217 (<0.1) |
|  | **Any atypical APD[[2]](#footnote-3)** | **2,596,440 (87.3)** |
| **Typical APD** | CHLORPROMAZINE HCL | 23,292 (0.8) |
|  | FLUPENTIXOL DECANOATE | 25,344 (0.9) |
|  | FLUPENTIXOL HCL | 7,659 (0.3) |
|  | FLUPHENAZINE DECANOATE | 7,549 (0.3) |
|  | FLUPHENAZINE HCL | 1,346 (0) |
|  | FLUSPIRILENE | 46 (<0.1) |
|  | HALOPERIDOL | 30,712 (1) |
|  | HALOPERIDOL DECANOATE | 3,480 (0.1) |
|  | HALOPERIDOL LACTATE | 128 (<0.1) |
|  | LOXAPINE HCL | 652 (<0.1) |
|  | LOXAPINE SUCCINATE | 130,385 (4.4) |
|  | MESORIDAZINE BESYLATE | 98 (<0.1) |
|  | METHOTRIMEPRAZINE HCL | 85 (<0.1) |
|  | METHOTRIMEPRAZINE MALEATE | 84,589 (2.8) |
|  | PERICYAZINE | 489 (<0.1) |
|  | PERPHENAZINE | 4,668 (0.2) |
|  | PIMOZIDE | 4,972 (0.2) |
|  | PIPOTIAZINE PALMITATE | 4,903 (0.2) |
|  | THIORIDAZINE HCL | 4,624 (0.2) |
|  | TRIFLUOPERAZINE HCL | 16,140 (0.5) |
|  | ZUCLOPENTHIXOL ACETATE | 125 (<0.1) |
|  | ZUCLOPENTHIXOL DECANOATE | 19,126 (0.6) |
|  | ZUCLOPENTHIXOL HCL | 5,903 (0.2) |
|  | FLUPHENAZINE ENANTHATE | 5 (<0.1) |
|  | THIOTHIXENE | 106 (<0.1) |
|  | **Any typical APD[[3]](#footnote-4)** | **376,426 (12.7)** |

**Supplementary Table 2: Offences committed during the study period (n=11,462)**

|  | **Charge description** | **# of offences n (%)** |
| --- | --- | --- |
| **Violent offences** | MURDER | 27 (0.3) |
|  | ATTEMPTED MURDER | 3 (0.1) |
|  | ROBBERY | 823 (9.5) |
|  | SEXUAL ASSAULT | 185 (2.1) |
|  | OTHER SEXUAL-RELATED OFFENCES | 268 (3.1) |
|  | MAJOR ASSAULT | 1,542 (17.9) |
|  | COMMON ASSAULT | 3,375 (39.2) |
|  | UTTERING THREATS | 2,008 (23.3) |
|  | CRIMINAL HARASSMENT | 244 (2.8) |
|  | OTHER CRIMES AGAINST A PERSON | 144 (1.7) |
|  | **Violent offences (total)** | **8,619 (100%)** |
| **Non-violent offences** | MOTOR VEHCLE THEFT | 246 (0.7) |
|  | THEFT | 10,242 (29.5) |
|  | BREAK & ENTER | 1,199 (3.5) |
|  | FRAUD | 1,433 (4.1) |
|  | MISCHIEF | 2,241 (6.5) |
|  | POSSESSION OF STOLEN PROPERTY | 1,280 (3.7) |
|  | OTHER PROPERTY CRIME | 295 (0.8) |
|  | FAILURE TO APPEAR IN COURT | 439 (1.3) |
|  | BREACH OF PROBATION | 6,352 (18.3) |
|  | UNLAWFULLY AT LARGE | 106 (0.3) |
|  | FAILURE TO COMPLY WITH ORDER | 4,306 (12.4) |
|  | OTHER ADMINISTRATIVE JUSTICE | 646 (1.9) |
|  | WEAPONS | 644 (1.9) |
|  | PROSTITUTION | 160 (0.5) |
|  | DISTURBING THE PEACE | 427 (1.2) |
|  | RESIDUAL CRIMINAL CODE | 1,608 (4.6) |
|  | IMPAIRED DRIVING | 196 (0.6) |
|  | OTHER CRIMINAL CODE TRAFFIC | 443 (1.3) |
|  | DRUG POSSESSION | 906 (2.6) |
|  | DRUG TRAFFICKING | 900 (2.6) |
|  | YOUNG OFFENDERS ACT | 17 (<0.1) |
|  | OTHER FEDERAL STATUTES | 238 (0.7) |
|  | OTHER | 409 (1.2) |
|  | **Non-violent offences (total)** | **34,733 (100%)** |

**Supplementary Table 3: Complete case analysis estimating the association between medication possession ratio and violent/non-violent crime (n=10,182[[4]](#footnote-5))**

|  | **Medication Possession Ratio (MPR)** | **Adjusted Rate Ratio; (95% CI)[[5]](#footnote-6)** |
| --- | --- | --- |
| Violent offences | MPR |  |
|  | ≤0.19 | **1.39 (1.27, 1.53)** |
|  | 0.20 - 0.39 | **2.28 (2.02, 2.58)** |
|  | 0.40 - 0.59 | **2.26 (1.99, 2.56)** |
|  | 0.60 - 0.79 | **1.78 (1.58, 2.00)** |
|  | ≥0.80 | Reference |
|  | MPR (<0.80) | **1.59 (1.46, 1.73)** |
| Non-violent offences | MPR |  |
|  | ≤0.19 | **1.30 (1.21, 1.41)** |
|  | 0.20 - 0.39 | **1.67 (1.52, 1.84)** |
|  | 0.40 - 0.59 | **1.60 (1.46, 1.75)** |
|  | 0.60 - 0.79 | **1.48 (1.36, 1.60)** |
|  | ≥0.80 | Reference |
|  | MPR (<0.80) | **1.41 (1.32, 1.50)** |

CI: Confidence Interval; MPR: Medication Possession Ratio

**Supplementary Table 4: Frequency and strength of atypical antipsychotic drugs (n=2,596,440)** prescribed to participants (n=11,462) during the study period

| **Generic name** | **Strength and formulation** | **N (%)[[6]](#footnote-7)** |
| --- | --- | --- |
| **ARIPIPRAZOLE (n=63,700[[7]](#footnote-8))** | 2 MG TABLET | 7,469 (11.7) |
|  | 5 MG TABLET | 14,533 (22.8) |
|  | 10 MG TABLET | 15,406 (24.2) |
|  | 15 MG TABLET | 15,241 (23.9) |
|  | 20 MG TABLET | 5,107 (8) |
|  | 30 MG TABLET | 5,902 (9.3) |
|  | 300 MG VIAL | 2 (<0.1) |
|  | 400 MG VIAL | 40 (0.1) |
| **ASENAPINE MALEATE (n=1,612)** | 5 MG TAB SUBLINGUAL | 580 (36) |
|  | 10 MG TAB SUBLINGUAL | 1,032 (64) |
| **CLOZAPINE (n=203,508)** | 25 MG TABLET | 63,559 (31.2) |
|  | 50 MG TABLET | 5,737 (2.8) |
|  | 100 MG TABLET | 129,550 (63.7) |
|  | 200 MG TABLET | 4,662 (2.3) |
| **OLANZAPINE (n=563,920)** | 2.5 MG TABLET | 32,595 (5.8) |
|  | 5 MG TAB RAPDIS | 47,116 (8.4) |
|  | 5 MG TABLET | 10,6955 (19) |
|  | 7.5 MG TABLET | 2,0507 (3.6) |
|  | 10 MG TAB RAPDIS | 89,850 (15.9) |
|  | 10 MG TABLET | 196,456 (34.8) |
|  | 10 MG VIAL | 1 (<0.1) |
|  | 15 MG TAB RAPDIS | 2,4547 (4.4) |
|  | 15 MG TABLET | 30,080 (5.3) |
|  | 20 MG TAB RAPDIS | 12,938 (2.3) |
|  | 20 MG TABLET | 2,875 (0.5) |
| **PALIPERIDONE (n=11,280)** | 3 MG TAB ER 24 | 5,817 (51.6) |
|  | 6 MG TAB ER 24 | 3,961 (35.1) |
|  | 9 MG TAB ER 24 | 1,502 (13.3) |
| **PALIPERIDONE PALMITATE (n=18,378)** | *SYRINGE[[8]](#footnote-9)* | 18,378 (100) |
| **QUETIAPINE FUMARATE (n=114,7367)** | 25 MG TABLET | 465,245 (40.5) |
|  | 50 MG TAB ER 24H | 38,168 (3.3) |
|  | 50 MG TABLET | 2,999 (0.3) |
|  | 100 MG TABLET | 323,101 (28.2) |
|  | 150 MG TAB ER 24H | 11,595 (1) |
|  | 150 MG TABLET | 4,736 (0.4) |
|  | 200 MG TAB ER 24H | 22,245 (1.9) |
|  | 200 MG TABLET | 155,736 (13.6) |
|  | 300 MG TAB ER 24H | 31,892 (2.8) |
|  | 300 MG TABLET | 80,179 (7) |
|  | 400 MG TAB ER 24H | 11,471 (1) |
| **RISPERIDONE (n=518,197)** | 1 MG TAB RAPDIS | 7,084 (1.4) |
|  | 1 MG TABLET | 176,879 (34.1) |
|  | 2 MG TAB RAPDIS | 9,992 (1.9) |
|  | 2 MG TABLET | 149,380 (28.8) |
|  | 3 MG TAB RAPDIS | 1,065 (0.2) |
|  | 3 MG TABLET | 67,959 (13.1) |
|  | 4 MG TAB RAPDIS | 339 (0.1) |
|  | 4 MG TABLET | 29,836 (5.8) |
|  | 5 MG TABLET | 2 (<0.1) |
|  | .25 MG TABLET | 13,129 (2.5) |
|  | .5 MG TAB RAPDIS | 3,071 (0.6) |
|  | .5 MG TABLET | 55,357 (10.7) |
|  | *SOLUTION* | 4,104 (0.8) |
| **RISPERIDONE MICROSPHERES** | *SYRINGE* | 43,352 (100) |
| **ZIPRASIDONE HCL (n=24,909)** | 20 MG CAPSULE | 4,220 (16.9) |
|  | 40 MG CAPSULE | 7,712 (31) |
|  | 60 MG CAPSULE | 4,767 (19.1) |
|  | 80 MG CAPSULE | 8,210 (33) |
| **LURASIDONE HCL (n=217)** | 20 MG TABLET | 53 (24.4) |
|  | 40 MG TABLET | 58 (26.7) |
|  | 60 MG TABLET | 3 (1.4) |
|  | 80 MG TABLET | 103 (47.5) |

24H: 24 HOURS; ER: EMERGENCY RELEASE; TAB: TABLET

**Supplementary Table 5: Frequency and strength of typical antipsychotic drugs (n=376,426) prescribed to participants (n=11,462) during the study period**

| **Generic name** | **Strength and formulation** | **N (%)[[9]](#footnote-10)** |
| --- | --- | --- |
| **CHLORPROMAZINE HCL (n=23,292[[10]](#footnote-11))** | *AMPUL[[11]](#footnote-12)* | 29 (0.1) |
|  | *DROPS* | 1 (<0.1) |
|  | *LIQUID* | 297 (1.3) |
|  | *SYRUP* | 4 (<0.1) |
|  | 10 MG TABLET | 111 (0.5) |
|  | 25 MG TABLET | 7,709 (33.1) |
|  | 50 MG TABLET | 8,468 (36.4) |
|  | 100 MG SUPP.RECT | 5 (<0.1) |
|  | 100 MG TABLET | 6,641 (28.5) |
|  | 200 MG TABLET | 27 (0.1) |
| **FLUPENTIXOL DECANOATE (n=25,344)** | *AMPUL* | 8,504 (33.6) |
|  | *VIAL* | 16,840 (66.4) |
| **FLUPENTIXOL HCL (n=7,659)** | 3 MG TABLET | 4,143 (54.1) |
|  | .5 MG TABLET | 3,516 (45.9) |
| **FLUPHENAZINE DECANOATE (n=7,549)** | *AMPUL* | 2,916 (38.6) |
|  | *VIAL* | 4,633 (61.4) |
| **FLUPHENAZINE HCL (n=1,346)** | 1 MG TABLET | 478 (35.5) |
|  | 2 MG TABLET | 253 (18.8) |
|  | 5 MG TABLET | 615 (45.7) |
| **FLUSPIRILENE (n=46)** | *AMPUL* | 30 (65.2) |
|  | *VIAL* | 16 (34.8) |
| **HALOPERIDOL (n=30,712)** | 1 MG TABLET | 5,525 (18) |
|  | 2 MG TABLET | 6,159 (20.1) |
|  | 5 MG TABLET | 12,907 (42) |
|  | .5 MG TABLET | 1,754 (5.7) |
|  | *AMPUL* | 119 (0.4) |
|  | *VIAL* | 3 (<0.1) |
|  | 10 MG TABLET | 4,146 (13.5) |
|  | 20 MG TABLET | 99 (0.3) |
| **HALOPERIDOL DECANOATE (n=3,480)** | *VIAL* | 3,480 (100) |
| **HALOPERIDOL LACTATE (n=128)** | *AMPUL* | 5 (3.9) |
|  | *ORAL CONC* | 123 (96.1) |
| **LOXAPINE HCL (n=652)** | *AMPUL* | 296 (45.4) |
|  | *ORAL CONC* | 356 (54.6) |
| **LOXAPINE SUCCINATE (n=130,385)** | 2.5 MG TABLET | 1,650 (1.3) |
|  | 5 MG TABLET | 28,861 (22.1) |
|  | 10 MG TABLET | 57,999 (44.5) |
|  | 25 MG TABLET | 32,093 (24.6) |
|  | 50 MG TABLET | 9,782 (7.5) |
| **MESORIDAZINE BESYLATE (n=98)** | 10 MG TABLET | 47 (48) |
|  | 25 MG TABLET | 45 (45.9) |
|  | 50 MG TABLET | 6 (6.1) |
| **METHOTRIMEPRAZINE HCL (n=85)** | *AMPUL* | 16 (18.8) |
|  | *SOLUTION* | 69 (81.2) |
| **METHOTRIMEPRAZINE MALEATE (n=84,589)** | 2 MG TABLET | 729 (0.9) |
|  | 5 MG TABLET | 19,525 (23.1) |
|  | 25 MG TABLET | 4,4541 (52.7) |
|  | 50 MG TABLET | 19,794 (23.4) |
| **PERICYAZINE (n=489)** | 5 MG CAPSULE | 96 (19.6) |
|  | *DROPS* | 11 (2.2) |
|  | 10 MG CAPSULE | 312 (63.8) |
|  | 20 MG CAPSULE | 70 (14.3) |
| **PERPHENAZINE (n=4,668)** | 2 MG TABLET | 1,229 (26.3) |
|  | 4 MG TABLET | 1,955 (41.9) |
|  | 8 MG TABLET | 1,216 (26) |
|  | 16 MG TABLET | 268 (5.7) |
| **PIMOZIDE (n=4,972)** | 2 MG TABLET | 3,361 (67.6) |
|  | 4 MG TABLET | 1,582 (31.8) |
|  | 10 MG TABLET | 29 (0.6) |
| **PIPOTIAZINE PALMITATE (n=4,903)** | *AMPUL* | 4,903 (100) |
| **THIORIDAZINE HCL (n=4,624)** | *ORAL CONC* | 25 (0.5) |
|  | *ORAL SUSP* | 57 (1.2) |
|  | 10 MG TABLET | 715 (15.5) |
|  | 25 MG TABLET | 1,345 (29.1) |
|  | 50 MG TABLET | 1,745 (37.7) |
|  | 100 MG TABLET | 737 (15.9) |
| **TRIFLUOPERAZINE HCL (n=16,140)** | 1 MG TABLET | 1,029 (6.4) |
|  | 2 MG TABLET | 4,672 (28.9) |
|  | 5 MG TABLET | 8,531 (52.9) |
|  | 10 MG TABLET | 1,682 (10.4) |
|  | 20 MG TABLET | 226 (1.4) |
| **ZUCLOPENTHIXOL ACETATE (n=125)** | *AMPUL* | 125 (100) |
| **ZUCLOPENTHIXOL DECANOATE (n=19,126)** | *AMPUL* | 19,126 (100) |
| **ZUCLOPENTHIXOL HCL (n=5,903)** | 10 MG TABLET | 4,948 (83.8) |
|  | 25 MG TABLET | 953 (16.1) |
|  | 40 MG TABLET | 2 (<0.1) |
| **FLUPHENAZINE ENANTHATE (n=5)** | *VIAL* | 5 (100) |
| **THIOTHIXENE (n=106)** | 2 MG CAPSULE | 57 (53.8) |
|  | 5 MG CAPSULE | 3 (2.8) |
|  | 10 MG CAPSULE | 46 (43.4) |

24H: 24 HOURS; AMPUL: AMPULE; CONC: CONCENTRATE; ER: EMERGENCY RELEASE; SUPP.RECT : SUPPOSITORY RECTAL; SUS: SUSPENSION; TAB: TABLET

1. -n represents total pharmacy transactions attributable to a specific antipsychotic and % represents the proportion of pharmacy transactions for a given antipsychotic against all antipsychotic-related pharmacy transactions (2,972,866) prescribed to participants during the study period. [↑](#footnote-ref-2)
2. -Includes all aforementioned (n=11) atypical antipsychotic drugs. [↑](#footnote-ref-3)
3. - Includes all aforementioned (n=25) typical antipsychotic drugs. [↑](#footnote-ref-4)
4. -1,280 participants (11%) were excluded from the analysis due to missing demographic information: age n=3 (<1%); ethnicity n=573 (5%); and education level n=1,193 (10%). [↑](#footnote-ref-5)
5. -Each multivariable GEE model controlled for age at enrolment (centered, age -18); gender (men & women); ethnicity (White, Indigenous and Other); education level (<Gd. 10, Gd. 10/11, Gd. 12 and Vocational/University); use of substance disorder-related services (continuous variable); number of offences in the previous year (continuous variable); number of 120-day intervals (continuous variable); and duration of follow up in days (offset variable). [↑](#footnote-ref-6)
6. -Percentages in parentheses (in this column) indicate the relative proportion of pharmacy encounters related to a specific drug among participants during the study period. [↑](#footnote-ref-7)
7. -Values in parentheses (in this column) indicate the number of pharmacy encounters related to a specific drug among participants during the study period. [↑](#footnote-ref-8)
8. -Italics indicate that strength information was unavailable. [↑](#footnote-ref-9)
9. -Percentages in parentheses (in this column) indicate the relative proportion of pharmacy encounters related to a specific drug. [↑](#footnote-ref-10)
10. -Values in parentheses (in this column) indicate the number of pharmacy encounters related to a specific drug. [↑](#footnote-ref-11)
11. -Italics indicate that drug strength information was unavailable. [↑](#footnote-ref-12)
